# Remote Monitoring of Patients With Hematologic Malignancies at High Risk of Febrile Neutropenia: Exploratory Study

**DOI:** 10.2196/33265

**Published:** 2022-01-25

**Authors:** Maxwell Kroloff, Ramin Ramezani, Holly Wilhalme, Arash Naeim

**Affiliations:** 1 UCLA Jonsson Comprehensive Cancer Center David Geffen School of Medicine University of California, Los Angeles Los Angeles, CA United States; 2 Center for Smart Health University of California, Los Angeles Los Angeles, CA United States; 3 Department of Computer Science University of California, Los Angeles Los Angeles, CA United States; 4 Division of General Internal Medicine and Health Services Research University of California, Los Angeles Los Angeles, CA United States

**Keywords:** remote monitoring, febrile neutropenia, temperature, heart rate, oxygen saturation, mobile health, mHealth, hematologic malignancies, hematology, malignancies, digital health, clinical outcomes

## Abstract

**Background:**

Febrile neutropenia is one of the most common oncologic emergencies and is associated with significant, preventable morbidity and mortality. Most patients who experience a febrile neutropenia episode are hospitalized, resulting in significant economic cost.

**Objective:**

This exploratory study implemented a remote monitoring system comprising a digital infrared thermometer and a pulse oximeter with the capability to notify providers in real time of abnormalities in vital signs that could suggest early clinical deterioration and thereby improve clinical outcomes.

**Methods:**

The remote monitoring system was implemented and compared to standard-of-care vital signs monitoring in hospitalized patients with underlying hematologic malignancies complicated by a febrile neutropenia episode in order to assess the feasibility and validity of the system. Statistical analysis was performed using the intraclass correlation coefficient (ICC) to assess the consistency between the measurements taken using traditional methods and those taken with the remote monitoring system for each of the vital sign parameters (temperature, heart rate, and oxygen saturation). A linear mixed-effects model with a random subject effect was used to estimate the variance components. Bland-Altman plots were created for the parameters to further delineate the direction of any occurring bias.

**Results:**

A total of 23 patients were enrolled in the study (mean age 56, SD 23-75 years; male patients: n=11, 47.8%). ICC analysis confirmed the high repeatability and accuracy of the heart rate assessment (ICC=0.856), acting as a supplement to remote temperature assessment. While the sensitivity and specificity for capturing tachycardia above a rate of 100 bpm were excellent (88% and 97%, respectively), the sensitivity of the remote monitoring system in capturing temperatures >37.8 °C and oxygen saturation <92% was 45% and 50%, respectively.

**Conclusions:**

Overall, this novel approach using temperature, heart rate, and oxygen saturation assessments successfully provided real-time, clinically valuable feedback to providers. While temperature and oxygen saturation assessments lagged in terms of sensitivity compared to a standard in-hospital system, the heart rate assessment provided highly accurate complementary data. As a whole, the system provided additional information that can be applied to a clinically vulnerable population. By transitioning its application to high-risk patients in the outpatient setting, this system can help prevent additional use of health care services through early provider intervention and potentially improve outcomes.

## Introduction

Febrile neutropenia is one of the most common oncologic emergencies, accounting for approximately 5% of all cancer-related hospitalizations [1]. Moreover, it is associated with significant preventable complications including hypotension, acute renal failure, heart failure, and early mortality, as well as substantial economic cost [2,3]. Across hematologic malignancies, the risk of febrile neutropenia, and, thus, additional morbidity and mortality, is further magnified. In fact, in a prospective observational study of 120 patients with acute myeloid leukemia (AML) and acute lymphocytic leukemia (ALL) undergoing induction chemotherapy, those who became neutropenic (n=107) also experienced at least one febrile neutropenia episode (FNE) [4]. In more novel treatment regimens such as engineered cell therapies, the rate of neutropenic fever remains exceedingly high, with a recent observational analysis of 60 patients confirming an incidence of 86.7% within the first 30 days after chimeric antigen receptor (CAR) T-cell infusion [5].

Despite current guidelines and multiple risk calculators to assist in risk stratification, the majority (up to 94%) of patients with febrile neutropenia continue to be admitted to the hospital, with an even larger percentage of patients being admitted at academic centers [3,6-8]. In the era of ever-increasing health care costs, hospitalizations related to febrile neutropenia result in an excess of $2.3 billion per year among adults and account for roughly 8% of all cancer-related costs [1]. Presumably, the persistently high rate of hospitalization is due to difficulty in risk stratification and the simultaneously high rate of preventable morbidity and mortality within this population. Thus, patients with febrile neutropenia represent a group in which there is ample opportunity for improved efficiency of care and more appropriate utilization of limited health care resources.

Recent advances in technology have made it possible to monitor a patient’s key vital signs outside of a clinic or hospital setting. Within the field of oncology, these devices are being increasingly integrated into clinical care and even into oncology trials, but the great majority of remote monitoring has focused on activity, sleep, and heart rate [9-11]. Only recently have studies begun to assess the efficacy and validity of either continuous or intermittent remote temperature monitoring in neutropenic patients, but these studies have unilaterally assessed temperature via skin patch [12,13]. Our exploratory study aimed to build upon a previous remote monitoring platform developed by the UCLA Center for SMART Health by incorporating a digital infrared thermometer and a pulse oximeter with the capability to notify providers in real time of abnormalities in vital signs that could suggest early clinical deterioration. The modified mobile health platform was subsequently implemented and compared to standard-of-care monitoring in hospitalized patients with underlying hematologic malignancies complicated by an FNE in order to assess the feasibility and validity of the system.

## Methods

### Remote System Development

This institutional review board–approved (IRB#20-000303) exploratory study built upon the previously developed Sensing At-Risk Patients (SARP) platform, which consisted of a smartwatch, a software application, and a central data processing and analytics engine [14-16]. The SARP platform was initially developed to remotely monitor elderly and at-risk patients in rehabilitation facilities and at home, focusing on activity monitoring. Building up this previously validated platform, a digital infrared thermometer (AndesFit Bluetooth 4.0 Wireless Non-Contact Infrared Body/Surface Thermometer, ADF-B38A, AndesFit Health) and a pulse oximeter (AndesFit Bluetooth 4.0 Pulse Oximeter, ADF-B06, AndesFit Health) with the capability to measure heart rate and oxygen saturation were incorporated into the system to review data remotely in real time via the HIPAA (Health Insurance Portability and Accountability Act)-compliant SARP website.

### Recruitment

We recruited patients aged 18 years or older with underlying leukemia or lymphoma admitted to the inpatient service at an academic tertiary care hospital whose course was complicated by an FNE. This included patients with newly diagnosed or relapsed/refractory disease, as well as those undergoing autologous or allogeneic stem cell transplants or CAR T-cell therapy. An FNE was defined as a temperature >38.3 °C or 38.0 °C sustained over 1 hour and a concurrent neutrophil count <500 cells/µL [17]. Patients were excluded if they were unable to comply with the additional monitoring for any reason.

The remote monitoring was implemented for 72 hours starting at the time of consent, which occurred within 24 hours of the patient’s most recent FNE. Once the participant enrolled in the study, they were given the remote monitoring system, which included the thermometer, pulse oximeter, and tablet. The system remained at the patient’s bedside for the remainder of the study. The health care staff, including the nursing staff and medical assistants, were instructed on the appropriate use of the remote monitoring system and performed all vital signs assessments. This instruction included an initial in-person group tutorial followed by one-on-one training and a demonstration at the time of implementation. The remote temperature and pulse oximeter assessments immediately followed the vital signs assessment using the standard hospital equipment (Phillips IntelliVue MX450), which occurred every 4 hours. At the end of the study period, the thermometer, pulse oximeter, and tablet were collected, charged, sanitized, and redistributed by the study team. The temperature, heart rate, and oxygen saturation data obtained by the health care staff every 4 hours were transmitted via Bluetooth to the tablet, which was connected by Wi-Fi and securely transmitted in real time to a secure HIPAA-compliant server. These data were automatically deidentified and could be reviewed using unique patient identifiers on the SARP application website.

### Statistical Analysis

Statistical analysis was performed using the intraclass correlation coefficient (ICC) to assess the consistency between the measurements taken using traditional methods and those taken with the remote monitoring system for each of the vital sign parameters (temperature, heart rate, and oxygen saturation). A linear mixed-effects model with a random subject effect was used to estimate the variance components. The ICC was measured as the proportion of variance between subject measurements out of the total variance. Since measurements were taken multiple times while the patient was hospitalized, the model also included a fixed effect for time. Bland-Altman plots were created for temperature, heart rate, and oxygen saturation to further delineate the direction of any occurring bias, as well as to detect ranges where a larger difference between the two collection methods is seen. Given that subjects were monitored for up to 72 hours and measurements were taken approximately every 4 hours during the study, it was assumed that all patients would have at least 5 measurements. Therefore, a random sample of 30 patients was estimated to produce the 2-sided 95% CI widths with an estimated range of 0.2 when the ICC is 0.80 and 0.06 when the ICC is 0.95.

## Results

### Patient Characteristics

A total of 23 patients were enrolled in the study, of which 17 patients had a confirmed diagnosis of AML and the remainder had diagnoses including ALL, multiple myeloma, diffuse large B-cell lymphoma, and blastic plasmacytoid dendritic cell neoplasm (Table 1). Of the enrolled patients, 8 were newly diagnosed and were undergoing induction chemotherapy, 7 were receiving salvage chemotherapy for relapsed/refractory disease, and 6 were undergoing either an autologous or allogeneic stem cell transplant. Two patients had recently received CAR T-cell therapy.

**Table 1:** Patient characteristics.

Characteristic			Patients (N=23)
Age (years), median (range)			56 (23-75)
Sex (male), n (%)			11 (47.8)
**Primary malignancy, n (%)**			
	Acute myeloid leukemia	17 (73.9)	
	Multiple myeloma	2 (8.7)	
	Diffuse large B-cell lymphoma	2 (8.7)	
	Acute lymphocytic leukemia	1 (4.4)	
	Blastic plasmacytoid dendritic cell neoplasm	1 (4.4)	
**Disease status, n (%)**			
	Newly diagnosed	8 (34.8)	
	Relapsed/refractory	9 (39.1)	
	Remission	6 (26.1)	
**Treatment, n (%)**			
	Induction chemotherapy	8 (34.8)	
	Salvage chemotherapy	7 (30.4)	
	Allogeneic or autologous stem cell transplant	6 (26.1)	
	Chimeric antigen receptor T-cell	2 (8.7)	

### Sensitivity and Specificity Compared to the Standard System

Upon completion of the study, the standard hospital monitoring system captured 34 temperature assessments above a threshold of 37.8 °C whereas the remote monitoring system captured 18 assessments (Table 2). With respect to the pulse oximeter, only 6 assessments had less than a threshold of 92% oxygen saturation using the hospital vital signs equipment compared to 9 assessments with the remote monitoring system. Finally, the heart rate assessment produced 74 readings above a threshold heart rate of 100 bpm using the hospital equipment compared to 66 with the remote monitoring system. Overall, the sensitivity and specificity for capturing tachycardia above a rate of 100 bpm was 88% and 97%, respectively, using the remote system (Table 3). For temperature and oxygen saturation, the specificity was 97% and 96%, respectively. However, the sensitivity of the remote monitoring system with respect to temperature and oxygen saturation was 45% and 50%, respectively.

**Table 2:** Proportion of remotely obtained vital signs exceeding a defined threshold.

Parameter	Proportion exceeding threshold^a^			Agreement (%)
	Standard, n (%)	Remote, n (%)		
Temperature	34 (21)	18 (11)	88	
SpO_2_^b^	6 (3)	9 (5)	95	
Heart rate	74 (42)	66 (38)	93	

^a^For temperature, a threshold of ≥37.8 °C was considered a fever. For SpO_2_, a threshold <92% was used. For heart rate, a threshold of 100 bpm was used.

^b^SpO_2_: oxygen saturation.

**Table 3:** The specificity and sensitivity of the remote monitoring system compared to standard hospital monitoring.

Parameter	AUC^a,b^ (95% CI)	Sensitivity^c^ (%)	Specificity^d^ (%)
Temperature	0.898 (0.846-0.950)	45	97
SpO_2_^e^	0.964 (0.929-0.998)	50	96
Heart rate	0.990 (0.981-0.999)	88	97

^a^AUC: area under the curve.

^b^AUC was estimated using a mixed-effects logistic regression predicting temperature ≥37.8 °C and SpO_2_ <92% with a fixed effect for the SARP result (as a binary predictor) and a random subject effect to account for repeated measures.

^c^Sensitivity is the true positive rate (ie, the proportion of patients who had a fever or low SpO_2_ and were correctly identified as such).

^d^Specificity is the true negative rate (ie, the proportion of patients who did not have a fever or low SpO_2_ and were correctly identified as such).

^e^SpO_2_: oxygen saturation.

### ICC Analysis

**Figure 1:**
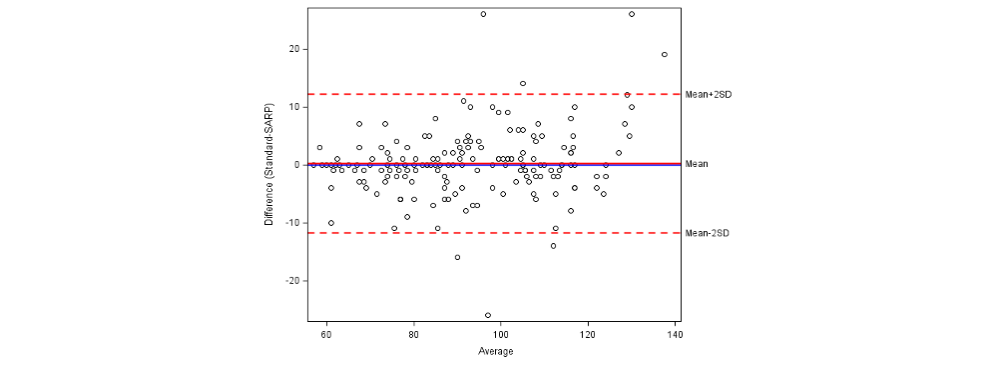
A Bland-Altman plot of heart rate agreement between the standard and remote monitoring methods. SARP: Sensing At-Risk Patients.

**Figure 2:**
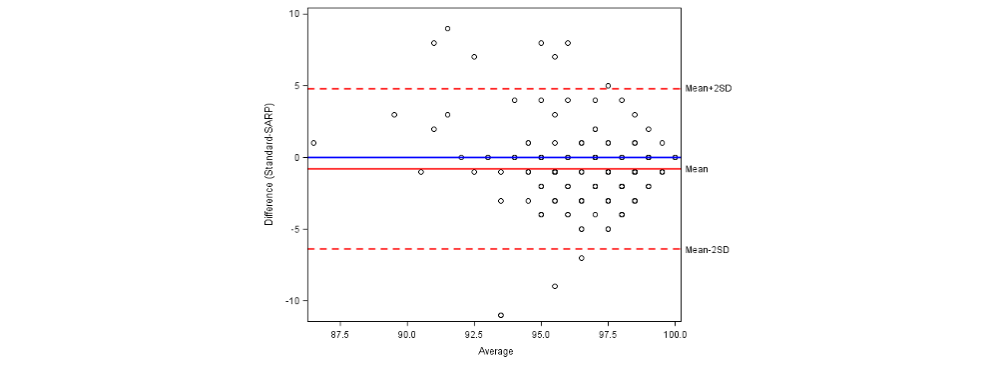
A Bland-Altman plot of oxygen saturation agreement between the standard and remote monitoring methods. SARP: Sensing At-Risk Patients.

**Figure 3:**
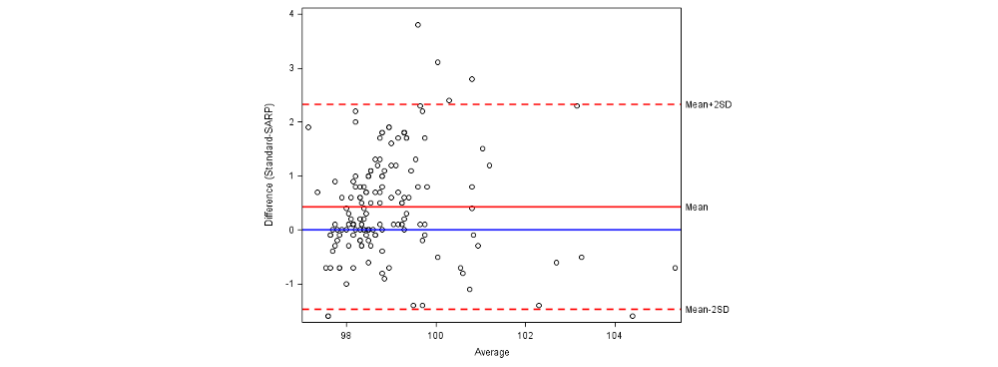
A Bland-Altman plot of temperature agreement between the standard and remote monitoring methods. SARP: Sensing At-Risk Patients.

The calculated ICC for heart rate was 0.856, which indicated that the repeatability between the standard and remote monitoring methods is excellent (Table 4). For oxygen saturation and temperature, the ICC was 0.233 and 0.363, respectively, indicating that the repeatability was significantly lower for the remote monitoring equipment. The Bland-Altman plots (Figures 1-3) further demonstrate repeatability by highlighting bias in the measurements obtained by the remote system compared to the standard equipment. While there was no clear bias in heart rate measurement, the remote temperature monitor was biased toward lower readings and the remote pulse oximeter was biased toward higher readings.

**Table 4:** Intraclass correlation coefficient (ICC) values comparing the standard system to remote monitoring.

Parameter	ICC^a^
Heart rate	0.856
SpO_2_^b^	0.233
Temperature	0.363

^a^ICC was obtained as the proportion of within-subject variance over the total variance. An intercept-only mixed-effects model was constructed with random effects for patient and for patient across the two methods to account for multiple observations per patient per method.

^b^SpO_2_: oxygen saturation.

### The Predictive Model

Finally, a predictive model was developed combining all 3 vital signs assessments in order to analyze the success of triggered alerts using the stated thresholds (heart rate >100 bpm, oxygen saturation <92%, temperature >37.8 °C) (Table 5). Again, the remote monitoring heart rate assessment produced an odds ratio of 248 compared to the standard in-hospital monitoring system, indicating the odds of measuring true tachycardia with the remote system. However, because there was substantial collinearity, only 4 models combining temperature, heart rate, and oxygen saturation converged, and additional combination did not significantly improve the predictability of any singular measure.

**Table 5:** A predictive model of alerts triggered.

Model and parameters^a^		Odds ratio (95% CI)	*P* value	AUC^b^ (95% CI)
**Model 1**				0.97 (0.94-0.99)
	Heart rate	248 (22-2896)	<.001	
	SpO_2_^c^	—^d^	—	
	Temperature	—	—	
**Model 2**				0.97 (0.94-0.99)
	Heart rate	268 (24-2992)	<.001	
	SpO_2_	6.1 (0.82-44.5)	.08	
	Temperature	—	—	
**Model 3**				0.95 (0.91-0.98)
	Heart rate	—	—	
	SpO_2_	—	—	
	Temperature	8.05 (1.16-55.9)	.04	
**Model 4**				0.95 (0.92-0.98)
	Heart rate	—	—	
	SpO_2_	2.2 (0.21-23.4)	.51	
	Temperature	7.2 (1.0-51.4)	.047	

^a^All variables were included as binary predictors (ie, cutoff at the thresholds that would trigger an alert). The model with all 3 variables did not converge.

^b^AUC: area under the curve.

^c^SpO_2_: oxygen saturation.

^d^Not available. Model convergence limited by multicollinearity.

## Discussion

### Principal Results

This exploratory pilot study demonstrated the feasibility of a self-monitoring system in an at-risk population while accurately providing multiple indicators of clinical status. In particular, the heart rate data alone were highly repeatable compared to a standard in-hospital heart rate assessment, as demonstrated by an ICC value of 0.865 and the correlation shown in the Bland-Altman plot. When using a cutoff analysis (heart rate >100 bpm), this finding was further corroborated, with a sensitivity and specificity of 88% and 97%, respectively; using a predictive model, the odds ratio was 248. These findings support both the repeatability of the remote heart rate data compared to standard in-hospital monitoring and the accuracy at which it can capture true tachycardia in an at-risk population. In combination with the temperature data, heart rate can serve as both a surrogate and adjunctive marker of clinical change, whether that be an FNE or an alternative, clinically significant change like dehydration requiring prompt intravenous fluid administration.

As a population, less than 40% of patients with an FNE may demonstrate concurrent tachycardia [18]. Within this cohort specifically, only 12 of the 17 true FNEs were associated with a heart rate >100 bpm. However, tachycardia associated with the FNE represented a high-risk feature, indicating possible clinical decline. In fact, in a multicenter prospective study of 346 patients with 515 FNEs, tachycardia at presentation was one of the strongest predictors of mortality [18]. The association was higher than all other abnormalities in vital signs, including tachypnea and hypotension, as well as many other known risk factors such as previous invasive fungal infections, oliguria, or initial positive blood cultures. Thus, the excellent performance of the heart rate monitor compared to standard inpatient monitoring represents additional, highly relevant clinical data which providers may use to early intervention, improving health outcomes and decreasing overall health care utilization.

### Comparison With Prior Work

Few studies have examined self- or remote monitoring in an at-risk cancer population and even fewer have prospectively assessed the implementation of wearable devices [19]. These studies have solely focused on continuous temperature monitoring in patients at high risk of neutropenia episodes. For instance, Dambrosio et al [20] used a continuous temperature skin patch on patients in the inpatient stem cell transplant unit and successfully demonstrated the repeatability of temperature assessment. Vera-Aguilera et al [12] went a step further by evaluating a wearable, continuous temperature monitor (tPatch) in patients undergoing autologous stem cell transplant in the outpatient setting. Measured febrile episodes were compared to self-measured oral temperatures taken every 3 to 4 hours using a standardized thermometer; the authors were able to demonstrate that the incidence of fever using the tPatch was 58.8% compared to 29.4% in the standard monitoring group. The success of these studies highlights the feasibility of remote temperature assessment. However, the unilateral assessment also leaves room for substantial improvement in optimizing patient care, especially given the predictive nature of other vital signs signifying potential clinical decline in an FNE. In comparison to these studies, by capturing a combination of temperature, heart rate, and oxygen saturation in a hospitalized, high-risk patient population, we were able to mirror a potential intervenable group, particularly in a postdischarge setting where the risk of readmission remains high. Furthermore, our novel approach provides additional variables beyond temperature monitoring, which can improve the likelihood of decreased health care utilization through early provider assessment and intervention prior to clinical decline.

### Limitations

With the spread of SARS-CoV-2 in 2019, and the resulting COVID-19 pandemic, there has been near-universal adoption of noncontact, infrared thermometers as an initial screening tool at the entrance of public spaces including hospitals, office buildings, retail stores, etc. These devices have largely been selected because of their availability, affordability, ease of use, and simultaneous noninvasive approach, which also prompted their inclusion in our remote monitoring system. Despite their many strengths including ease of use and patient familiarity, multiple studies have questioned their validity [21]. Admittedly, the digital infrared thermometer does lag in terms of repeatability when compared to the standard hospital monitor used in this analysis. Specifically, it has a relatively lower sensitivity in capturing FNEs, which does highlight some of the challenges of remote monitoring. Ideally, as technology improves, more reliable thermometers and pulse oximeters will limit these potential false negatives. Regardless, it is critical to emphasize that a remote monitoring system acts as a complement to traditional outpatient monitoring, which would typically consist of clinic visits with a singular assessment of vital signs upon presentation. While the additional data presents tremendous opportunity for improvement in clinical outcomes, the lack of a fever or hypoxia as captured by the remote system should not be interpreted as true absence of fever or hypoxia.

Although the remote temperature and oxygen assessment may miss some hypoxia or febrile episodes, it also has the potential to capture alternative markers of early clinical deterioration via incorporation of heart rate and oxygen saturation assessment. For instance, patients with cancer are more likely to be diagnosed with COVID-19 than the general population and more likely to have severe complications, such as intubation [22]. Beyond emphasizing the importance of vaccination in this patient population, monitoring temperature, heart rate, and oxygen saturation, provides additional opportunities to capture early infection and/or clinical deterioration, such as progressive hypoxia, which may precede intubation. Furthermore, additional objective assessments will likely enrich the understanding of COVID-19 among patients with cancer.

### Future Directions

After demonstrating the feasibility of this exploratory pilot study among inpatients, the critical next step is its implementation in a high-risk patient population in the outpatient setting, such as those who have recently been discharged after CAR T-cell therapy, those receiving outpatient autologous stem cell transplant, or those with prolonged neutropenia (eg, after consolidation with high-dose cytarabine). For reference, up to 32% of CAR T-cell recipients experience prolonged cytopenia of unclear etiology beyond day 28 and remain at substantially high risk of infection and subsequent rehospitalization [23]. Thus, applying the remote monitoring system to this group of patients with self-monitoring every 4 to 6 hours would provide real-time clinical indicators to providers with the capability to send alerts for abnormal vitals, such as a temperature >38 °C or a heart rate >100 bpm. In particular, because the sensitivity and specificity of the heart rate monitor is exceptional while the sensitivity of detecting hypoxia or fever lags, an alert set for tachycardia combined with either hypoxia or fever could specifically serve as a marker of clinical deterioration while limiting false alerts. The alert would then be sent to the designated provider who would have the flexibility to determine if the change in vital signs warrants a telephone call, in-person assessment, or neither. Such a protocol would further clarify whether tachycardia truly represents an FNE, whether the alerts are actionable, and most importantly, whether the alerts impact clinical outcomes.

### Conclusion

In summary, this exploratory study involving temperature, heart rate, and oxygen saturation assessments successfully provides real-time, clinically valuable feedback to providers. While the temperature and oxygen saturation lacked sensitivity when compared to a standard in-hospital system, the heart rate assessment provided highly accurate complementary data. As a whole, the system provided additional information that was applicable to a clinically vulnerable population. By transitioning its application to high-risk patients in the outpatient setting, our novel system can help prevent additional health care utilization through early provider intervention and potentially improve outcomes.

## Abbreviations

ALL: acute lymphocytic leukemia
AML: acute myeloid leukemia
CAR: chimeric antigen receptor
FNE: febrile neutropenia episode
HIPAA: Health Insurance Portability and Accountability Act
ICC: intraclass correlation coefficient
SARP: Sensing At-Risk Patients
